# Clinical outcomes of bovine pericardial tissue patch grafting for surgical correction of Peyronie’s disease: a prospective single-center case series

**DOI:** 10.1186/s12610-025-00298-7

**Published:** 2026-01-21

**Authors:** Pankaj M. Joshi, Pawan Kandhari, Meritxell Costa, Saurabh Thakkar, Emmanuel Oyibo, Shreyas Bhadranawar, Sanjay Kulkarni

**Affiliations:** 1 UroKul, Uro Surgery Institute, Pune, India; 2 Kulkarni Endosurgery Institute, Pune, India; 3 Department of Surgery, Federal Medical Centre Keffi, Urology Clinic, Keffi, Nasarawa State Nigeria

**Keywords:** Peyronie's disease, Bovine pericardium, Graft, Penile curvature, Surgical outcomes, Maladie de La Peyronie, Péricarde bovin, Greffe, Courbure pénienne, Résultats chirurgicaux

## Abstract

**Background:**

Surgical correction of Peyronie’s disease often requires grafting techniques to restore penile anatomy and function. The bovine pericardial patch is a promising biomaterial due to its strength, elasticity, and biocompatibility. This study evaluates the safety, handling, and clinical outcomes associated with its use in tunical grafting procedures.

**Methods:**

A prospective, single-centre clinical study enrolled six patients with Peyronie’s disease requiring plaque incision and grafting. Data on surgical handling characteristics, postoperative outcomes, complications, and patient satisfaction were collected and analysed descriptively using standard statistical methods.

**Results:**

All surgeries were completed without intraoperative complications. Patch handling characteristics were rated as excellent across all surgical parameters. No adverse events, graft-related complications, or loss of penile function were observed during the follow-up period. Patient satisfaction was rated as very high at discharge and at one-month follow-up.

**Conclusions:**

Bovine pericardial tissue patch grafting demonstrated excellent safety, efficacy, and high satisfaction rates for surgical correction of Peyronie’s disease deformities. These findings support its wider clinical application, although larger studies with extended follow-up are warranted to confirm long-term outcomes.

## Résumé

**Contexte:**

La correction chirurgicale de la maladie de La Peyronie nécessite souvent des techniques de greffe pour restaurer l’anatomie et la fonction péniennes. Le patch péricardique bovin est un biomatériau prometteur en raison de sa solidité, de son élasticité et de sa biocompatibilité. La présente étude a évalué la sécurité, le traitement et les résultats cliniques associés à son utilisation dans les procédures de greffe de la tunique. Une étude clinique prospective monocentrique a recruté six patients atteints de la maladie de La Peyronie nécessitant une incision et une greffe de plaques. Les données sur les caractéristiques du traitement chirurgical, les résultats postopératoires, les complications et la satisfaction des patients ont été collectées et analysées de manière descriptive à l’aide de méthodes statistiques standard.

**Résultats:**

Toutes les interventions chirurgicales ont été réalisées sans complications intra opératoires. Les caractéristiques de manipulation des patchs ont été jugées excellentes pour tous les paramètres chirurgicaux. Aucun événement indésirable, complication liée au greffon ou perte de fonction pénienne n’a été observé pendant la période de suivi. La satisfaction de chaque patient a été évaluée comme très élevée à la sortie et au suivi après un mois.

**Conclusions:**

La greffe de patch de tissu péricardique bovin a montré une excellente sécurité et efficacité ainsi que des taux élevés de satisfaction pour la correction chirurgicale des déformations de la maladie de La Peyronie. Ces résultats soutiennent une application clinique plus large, bien que des études incluant des effectifs plus importants avec un suivi plus prolongé soient justifiées pour confirmer les résultats à long terme.

## Introduction

Peyronie’s disease (PD) is a localised connective tissue disorder of the penile tunica albuginea, characterised by the development of fibrous plaques, which can lead to penile curvature, pain, and sexual dysfunction [1]. The prevalence of PD is estimated to range from 3% to 9% in the general male population, although it may be underreported due to patient reluctance [2, 3]. Surgical correction remains the gold standard for patients with severe deformities, failed medical management, or significant functional impairment [4]. 

Several surgical techniques exist for PD, including plication, plaque incision/excision with grafting, and implantation of penile prostheses [5]. Grafting is preferred in patients with significant curvature (> 60 degrees) or complex deformities where plication would result in unacceptable penile shortening [6]. The ideal graft material must be durable, flexible, biocompatible, non-immunogenic, and easy to handle intraoperatively [7]. 

Bovine pericardium has emerged as an attractive graft material due to its favourable biomechanical properties, high tensile strength, pliability, and ease of integration into host tissues [8, 9]. It has been widely used in cardiovascular and reconstructive surgeries, demonstrating excellent outcomes and minimal adverse events [10, 11]. Its application in penile surgery, although less extensively studied, has shown promising results [12, 13]. 

The choice of graft material critically impacts long-term outcomes, including recurrence of curvature, erectile dysfunction, graft contracture, and patient satisfaction [14]. Previous studies have evaluated autologous, synthetic, and xenogeneic grafts, but consensus regarding the optimal material remains elusive [5]. Bovine pericardium offers potential advantages over synthetic and some autologous options, including reduced surgical time, absence of donor site morbidity, and superior handling characteristics [15]. 

However, comprehensive prospective data evaluating the use of bovine pericardial patches specifically for tunical grafting in PD patients remain limited. This study aims to systematically assess the clinical performance, safety, and surgeon-reported handling characteristics of a bovine pericardial tissue patch in patients undergoing surgical correction for Peyronie’s disease. The primary outcomes included intraoperative and postoperative complication rates, postoperative penile function, and patient satisfaction.

We hypothesised that the bovine pericardial patch would demonstrate excellent integration, low complication rates, and high satisfaction amongst both surgeons and patients.

## Patients and methods

### Study design and population

A prospective, single-centre study was conducted enrolling six patients diagnosed with Peyronie’s disease, who required surgical correction using plaque incision and grafting techniques. Inclusion criteria included men aged 18 years and above with disabling penile curvature secondary to PD and willingness to participate and provide informed consent. Exclusion criteria included congenital curvature, complex deformities (hourglass deformity or constriction bands), requirement for prosthetic implantation, or known bovine product allergies.

### Surgical technique

**Fig. 1:**
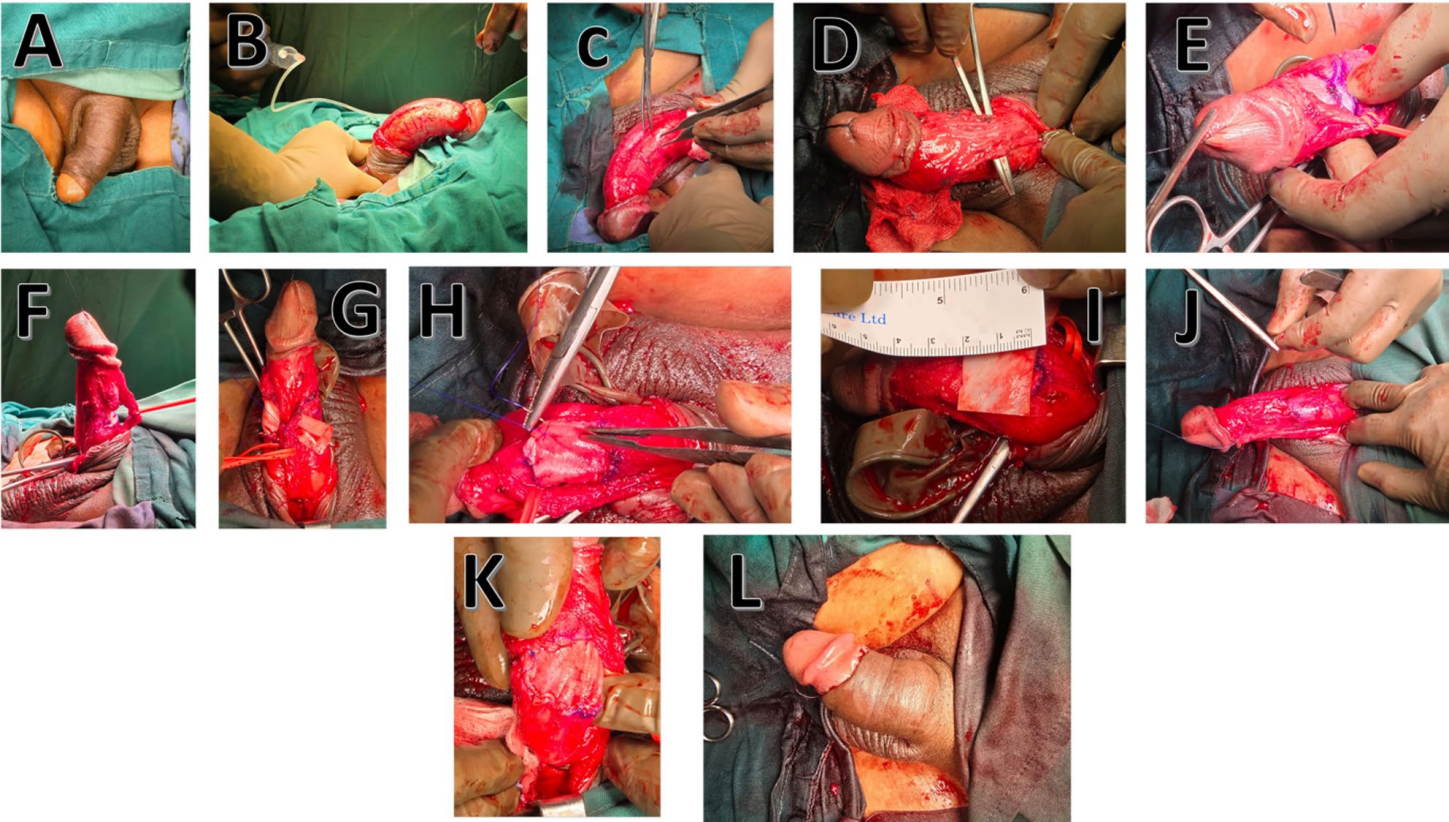
Surgical steps for plaque incision and grafting in Peyronie’s disease correction. This figure illustrates the sequential surgical technique employed for bovine pericardial patch grafting in Peyronie's disease correction. The procedure involves the following standardised steps:**A-B:** Artificial erection to assess degree and site of curvature.**C:** Subcoronal circumferential incision and complete penile degloving.**D:** Identification and marking of the point of maximal curvature.**E:** Dissection and mobilisation of the neurovascular bundle for dorsal plaques.**F-H:** Longitudinal incision of the tunica albuginea over the fibrotic plaque.**I:** Measurement of tunical defect created by plaque incision.**J:** Tailoring of bovine pericardial graft to fit the tunical defect.**K:** Suturing of graft to tunical edges with interrupted or running absorbable sutures.**L:** Repositioning of neurovascular bundle and verification of haemostasis. All procedures were performed by a single senior urological surgeon to ensure procedural consistency. The bovine pericardial patch (Invengenx-CR®, Tisgenx Inc, Irvine, CA, USA) demonstrated excellent handling characteristics throughout all surgical steps

Following standard aseptic protocols, plaque incision was performed under regional anaesthesia to correct the curvature. The defect was grafted with a bovine pericardial patch (Invengenx-CR^®^ patch manufactured by Tisgenx Inc, Irvine, CA, USA) tailored to the incision size and secured with interrupted absorbable sutures. All surgeries were performed by the same senior urological surgeon to ensure procedural consistency (Fig. 1).

### Data collection

Demographic data, intraoperative parameters (handling characteristics, operative time), hospital stay duration, postoperative complications, and adverse events were recorded. Patient satisfaction was assessed using a 5-point Likert scale [16]. 

### Follow-up

Postoperative assessments were done immediately after surgery and at 1 month.

### Statistical analysis

Given the small sample size, data were analysed descriptively. Continuous variables are presented as means and standard deviations, whilst categorical variables are expressed as frequencies and percentages. Statistical analyses were performed using Microsoft Excel 2016 (Microsoft Corporation, Redmond, WA, USA). Due to the exploratory nature of this study and the limited sample size (*n* = 6), formal hypothesis testing and p-values were not calculated; descriptive statistics were deemed most appropriate for summarising the data. Graphs were generated using standard spreadsheet software.

## Results

### Baseline characteristics

A total of six patients diagnosed with Peyronie’s disease were enrolled in the study. The baseline demographic details are presented in Table 1. The mean age of the patients was 40.4 ± 4.8 years, and the mean weight was 71.6 ± 1.1 kg. All patients were male.

**Table 1:** Baseline characteristics of the study population

Patient ID	Age (years)	Weight (kg)
1	32	76
2	46	76
3	29	68
4	47	66
5	48	72
6	39	91

This table presents the demographic characteristics of the six patients enrolled in the prospective study. Age and weight are individual patient measurements recorded at baseline assessment prior to surgical intervention

### Intraoperative outcomes

All surgeries were completed successfully following the standardised plaque incision and grafting procedure. No intraoperative complications were observed. The bovine pericardial patches demonstrated excellent handling characteristics across all cases. Surgical evaluations rated the grafts with perfect scores (10/10) for flexibility, elasticity, surgical seating, suture retention, and haemostasis. The grafts conformed well to the anatomical defects and maintained structural integrity throughout the procedures, without tearing or excessive bleeding.

### Postoperative outcomes

The mean duration of hospital stay was two days. None of the patients required admission to an intensive care unit. No postoperative complications were observed during the hospital stay or at follow-up assessments. Specifically, there were no instances of surgical site infections, necrosis, ischaemia, haematoma, thrombus formation, calcification, pseudoaneurysm formation, graft dehiscence, or recurrent penile curvature. Wound healing was satisfactory in all cases, and there were no reports of fibrosis or loss of penile sensation.

### Patient satisfaction

Patient satisfaction was assessed using a 5-point Likert scale immediately at discharge and again at the one-month follow-up. At the time of discharge, all six patients (100%) rated their satisfaction as “very satisfied.” At the one-month follow-up, all six patients continued to report “very satisfied” ratings. The patient satisfaction scores are summarised in Table 2.

**Table 2:** Patient satisfaction scores using 5-Point Likert scale

Timepoint	Very Satisfied	Satisfied	Neutral	Dissatisfied	Very Dissatisfied
Discharge	6	0	0	0	0
1 Month	6	0	0	0	0

Patient satisfaction was assessed using a validated 5-point Likert scale (1 = very dissatisfied, 2 = dissatisfied, 3 = neutral, 4 = satisfied, 5 = very satisfied) at two timepoints: immediately athospital discharge and at one-month postoperative follow-up. Data are presented as frequencies(number of patients) in each satisfaction category. All six patients (100%) reported the highestsatisfaction rating at both assessment points. Due to the absence of variability in responses, nostatistical comparison between timepoints was performed

## Discussion

This prospective study evaluated the clinical outcomes associated with the use of bovine pericardial tissue patches for plaque incision and grafting in patients with Peyronie’s disease. Our findings demonstrate that bovine pericardial grafts provide excellent surgical handling characteristics, facilitate satisfactory anatomical correction, and are associated with favourable short-term postoperative outcomes. Importantly, there were no intraoperative or postoperative complications, and patient satisfaction was uniformly high.

The surgical management of Peyronie’s disease typically depends on the severity of penile curvature, the presence of deformities such as hourglass deformities, and the patient’s sexual function. For patients with severe curvature, particularly those exceeding 60 degrees or with complex deformities, plaque incision and grafting (PIG) is the preferred approach [1, 2]. In this context, the choice of graft material plays a critical role in influencing both short- and long-term outcomes.

Bovine pericardium has several desirable properties as a graft material. It is characterised by high tensile strength, elasticity, biocompatibility, and resistance to infection [8, 9]. Unlike synthetic grafts, bovine pericardium induces minimal inflammatory response and integrates well with host tissue [10]. Compared to autologous grafts, such as saphenous vein or tunica vaginalis, it avoids the morbidity associated with graft harvesting and reduces operative time [11, 12]. 

Our study’s finding that the patch achieved perfect handling scores during surgery is consistent with previous reports from cardiovascular and urological surgeries where bovine pericardial patches were used [8, 13]. Ease of cutting, suturing, and tissue conformity are critical attributes that directly influence operative success, especially in delicate procedures such as penile reconstruction. Furthermore, the structural integrity of the patch remained uncompromised throughout the procedures, and there were no incidents of intraoperative tearing, which reflects its mechanical reliability.

The comparative xenograft study by Schlachtenberger et al. demonstrated that while both porcine and bovine pericardial patches elicited only a moderate and broadly similar early inflammatory response, bovine patches showed significantly greater microvascular ingrowth during the first two postoperative weeks, evidenced by higher CD31-positive microvessel density (23.2 ± 4.3 vs. 16.5 ± 5.8 mm², *P* = 0.001) and a nonsignificant trend toward higher functional capillary density. Neither material increased apoptosis or altered proliferative activity in adjacent tissues, suggesting equivalent short-term biocompatibility, but the superior neovascularization around bovine grafts implies earlier and more efficient incorporation into host tissue [17]. These observations are relevant to our Peyronie cohort because they highlight how structural and biochemical features of collagen-based biomaterials can substantially influence early integration, vascular remodeling, and host tissue response—parameters that are likewise central to the behaviour of tunical plaques and the performance of graft materials evaluated in our manuscript.

In the postoperative period, none of the patients developed infections, haematomas, graft-related necrosis, residual leaks, or calcifications. These outcomes compare favourably to results reported with other graft materials. Studies using dermal grafts, for example, have demonstrated postoperative complication rates ranging from 5% to 20%, including infections, fibrosis, and loss of penile sensation [14, 18]. The absence of any adverse events in our cohort supports the hypothesis that bovine pericardial patches offer a safer alternative.

Patient satisfaction, assessed via a Likert scale, was uniformly high. Satisfaction in Peyronie’s surgery is influenced by multiple factors, including correction of curvature, preservation of penile length, sexual function, and absence of complications [19]. All patients in our study reported being “very satisfied” both at discharge and at one-month follow-up, suggesting that the surgical and functional outcomes met or exceeded patient expectations.

### Limitations of the study

This study has several important limitations. Firstly, the small sample size (*n* = 6) substantially limits the statistical power and generalisability of our findings. Such a limited cohort precludes robust comparative analysis and increases the risk of type II error, wherein true differences in outcomes may remain undetected. Additionally, the absence of a control group utilising alternative graft materials prevents direct comparative assessment of efficacy and safety.

Secondly, the relatively short follow-up period of one month is insufficient to capture delayed complications or long-term outcomes. Peyronie’s disease is a chronic condition, and long-term follow-up is necessary to assess for delayed complications such as curvature recurrence, graft contracture, or erectile dysfunction [20, 21]. Future studies with larger sample sizes and longer follow-up durations are warranted to confirm the durability of these favourable outcomes and to validate the findings in more diverse populations.

Another limitation is the absence of a control group using an alternative graft material. Comparative studies would help establish whether bovine pericardial tissue confers superior outcomes relative to other biological or synthetic grafts. Randomised controlled trials comparing bovine pericardial patches with commonly used autologous and synthetic materials could provide more definitive evidence regarding its efficacy.

## Conclusion

This study provides preliminary evidence supporting the use of bovine pericardial tissue patches for tunical grafting in Peyronie’s disease. The material exhibited excellent intraoperative handling, a strong safety profile, and high short-term patient satisfaction. Given these promising results, bovine pericardial tissue appears to be a highly suitable option for plaque incision and grafting procedures in patients with Peyronie’s disease. Nonetheless, ongoing evaluation through larger and longer-term studies is essential to confirm these findings and to establish bovine pericardium as a standard graft material in urological reconstructive surgery.

## Data Availability

No datasets were generated or analysed during the current study.

## Abbreviations

PD: Peyronie’s disease
PIG: Plaque incision and grafting
